# A Sole Case of the FGF23 Gene Mutation c.202A>G (p.Thr68Ala) Associated with Multiple Severe Vascular Aneurysms and a Hyperphosphatemic Variant of Tumoral Calcinosis—A Case Report

**DOI:** 10.3390/life14050613

**Published:** 2024-05-10

**Authors:** Nevena Georgieva Ivanova

**Affiliations:** 1 Department of Urology and General Medicine, Faculty of Medicine, Medical University of Plovdiv, 4000 Plovdiv, Bulgaria; nevenai@yahoo.com; Tel.: +35-98-8913-0416; 2 St Karidad MHAT, Karidad Medical Health Center, 4004 Plovdiv, Bulgaria

**Keywords:** hyperphosphatemic tumoral calcinosis, vascular aneurysms, homozygous, new FGF23 mutation variant

## Abstract

Tumoral calcinosis is an extremely rare genetic disease caused by mutations in three genes, GALNT3, FGF23, and KL, which disrupt phosphorus metabolism. The hallmark of this condition is the formation of tumors in the soft tissues around the joints. Other phenotypic features of tumoral calcinosis are dental involvement and brain and vascular calcifications. The clinical case reported herein presents for the first time to the scientific community the c.202A>G (p.Thr68Ala) mutation of the FGF23 gene, associated with a hyperphosphatemic variant of tumoral calcinosis and multiple severe vascular aneurysms. A female patient underwent multiple surgeries for tumor formations in her soft tissues that first appeared at the age of 12 months. On this occurrence, the patient was found to have hyperphosphatemia, low phosphate clearance, increased tubular reabsorption with normal levels of total and ionized calcium, vitamin D3, and parathyroid hormone, and no effect of treatment with sevelamer hydrochloride and a low-phosphate diet. At the age of 39, the patient underwent imaging studies due to edema and a pulsating formation in the neck area, which revealed multiple vascular aneurysms with thrombosis, for which she received operative and interventional treatment. In this connection, and because of the established phosphorus metabolism disturbance, a genetic disease was suspected. The sequence analysis and deletion/duplication testing of the 358 genes performed on this occasion revealed that the woman was homozygous for a variant of the c.202A>G (p.Thr68Ala) mutation of the FGF23 gene. The established mutation is not present in population databases. The presented clinical case is the first and only one in the world to demonstrate the role of this type of FGF23 gene mutation in the development of a hyperphosphatemic variant of tumoral calcinosis characterized by aggressive formation of multiple vascular aneurysms.

## 1. Introduction

Tumoral calcinosis is a clinical and histopathological syndrome that was first defined in 1943 by Inclan et al. [1]. According to one of the proposed pathogenesis-based classifications, tumoral calcinosis has two major variants: the primary normophosphatemic subtype, which has normal calcium and phosphorus levels, and the primary hyperphosphatemic or familial subtype, which has normal calcium and high phosphorus levels [2]. The latter, which appears most frequently in the first or second decade of life [3], is an extremely rare genetic disorder, with fewer than 100 confirmed cases initially reported from Africa and the Middle East, then later from Europe [4,5].

The disease’s etiology is linked to inactivating mutations in the N-acetylgalactosaminyltransferase 3 gene (GalNAc transferase 3 gene, GALNT3), mutations in the fibroblast growth factor-23 (FGF23) gene, and membrane-bound protein Klotho (KL), which are inherited in an autosomal recessive manner, resulting in a defect in the function of fibroblast growth factor-23, a phosphaturic hormone [6,7], and thus a disruption in phosphorus regulation [8]. There is also a secondary variant of tumoral calcinosis, which is most often associated with chronic renal failure [2]. The main clinical manifestation of the two major variants involves the deposition of calcium salts in the soft tissues around the joints [9,10], most often in the upper limbs (shoulder and elbow), hip, foot, and wrist [11]. Tumors develop and can grow over time [12], sometimes becoming large enough to impair joint function [1,9]. The phosphaturic hormone FGF23, produced by osteoblasts, plays an important role in mineral metabolism by inhibiting the proximal tubular natrium/inorganic phosphorus-2a (Na/Pi-2a) and Na/Pi-2c cotransporters [13]. It has been suggested that its effects are mediated by its co-factor Klotho. Research in FGF23-null and Klotho knockout mice has found that the mice develop severe vascular calcinosis and hyperphosphatemia and have a reduced lifespan [14]. Additionally, there are studies demonstrating that there is a positive correlation between high levels of FGF23 and an increased risk of adverse cardiovascular events [15]. Moreover, there is even a report in the literature on familial tumoral calcinosis associated with cerebral and peripheral vascular aneurysms [16]. When the disease progresses, it may impact the teeth and jawbones [17,18], as well as lead to the formation of calcifications in various brain structures [19] and blood vessels, including the aorta, iliac, and carotids, as well as cerebral, coronary, and other vessels [20].

The clinical case I report presents to the scientific community for the first time the c.202A>G (p.Thr68Ala) mutation of the FGF23 gene, associated with a hyperphosphatemic variant of tumoral calcinosis and multiple severe vascular aneurysms with thrombosis.

## 2. Clinical Case Presentation

A 40-year-old female patient was readmitted to the orthopedics department yet another time due to a tumor formation in her right shoulder that continued to grow and, in the last week, began to cause pain in her shoulder joint, limiting movement and causing pain in the forearm. She reported at least 10 prior surgical interventions because of tumors developing in various areas (the left forearm, the proximal third of the left thigh, and the right deltoid), which had started as calcium deposits when she was 12 months old. The laboratory tests performed during these hospitalizations showed that the patient had a lot of problems with phosphorus metabolism, including hyperphosphatemia, low phosphate clearance, and increased tubular reabsorption at normal levels of total and ionized calcium, vitamin D3, and the parathyroid hormone. In 2011, the patient was admitted to the endocrinology department for further diagnostic evaluation. Several imaging studies were performed in search of pathological changes. A native X-ray of the palm and wrist revealed bone structure with higher density at the border between the distal epiphyses and the metaphyses of metacarpal bones 2, 3, 4, and 5, as well as a bilateral low-density bone structure in the carpal bones, the epiphyses of the metacarpal bones, and the phalanges. Areas of greater density and pseudocystic lucency were observed in the ribs and bodies of the involved vertebrae, as revealed by computed tomography of the neck and anterior mediastinum. Although the imaging and biochemical testing revealed some anomalies, no definitive diagnosis was reached, and the patient was discharged with a prescription for home therapy with 800 mg of sevelamer hydrochloride three times a day to lower serum phosphate levels and a diet plan. The treatment failed and was discontinued. The patient is currently taking no medication.

**Figure 1:**
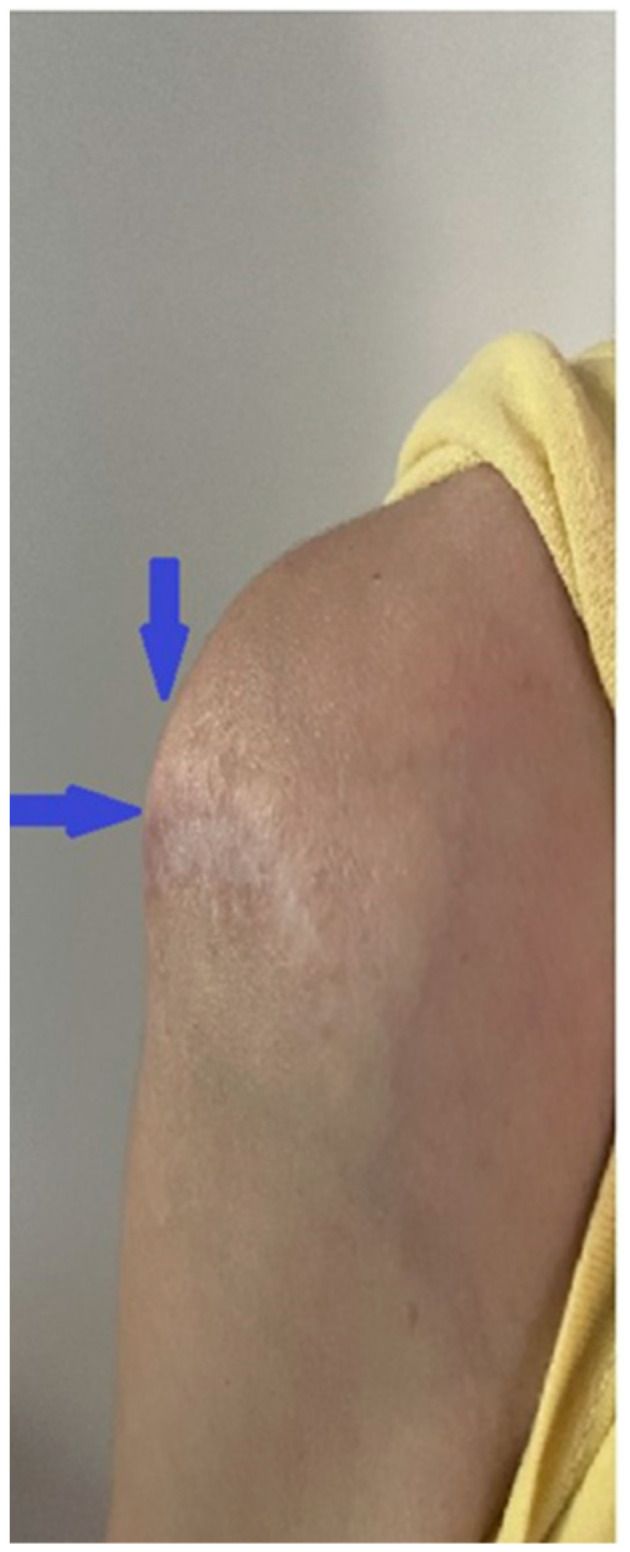
Image displaying a tumor encircling the right shoulder joint and a scar from a prior surgical intervention.

Upon examination during the patient’s current admission to the orthopedics department, a tumor mass was found in the right shoulder region (Figure 1).

**Figure 2:**
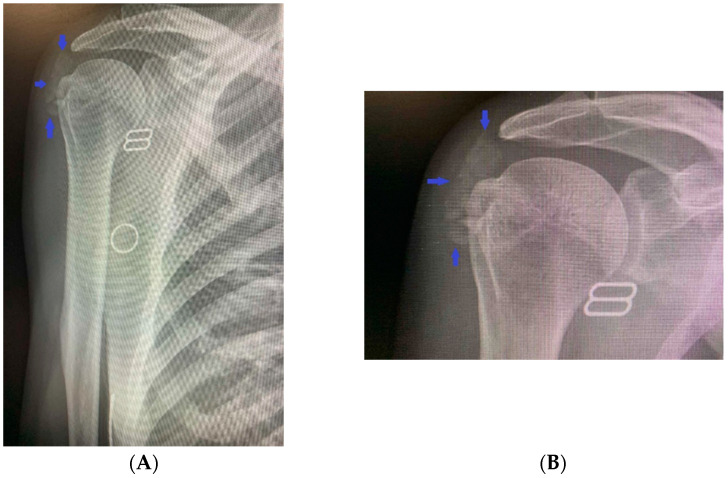
(**A**,**B**) An X-ray of the right shoulder joint revealing calcium deposits in the surrounding soft tissues.

Additionally, there was functional, palpable discomfort, along with movement limitations. The general physical status did not show any pathological abnormalities. Biochemical tests confirmed the previous findings of disturbances in the phosphorus metabolism of the patient: hyperphosphatemia (2.27 mmol/L, normal range 0.77–1.36 mmol/L), reduced 24 h urinary excretion (9.2 mmol/L, normal range 10.9–32.3 mmol/L), low 24 h phosphate clearance (0.08 mL/s, normal range 0.140–0.151 mL/s), increased tubular reabsorption (94.1%), and normal levels of total calcium (2.41 mmol/L, normal range 2.15–2.5 mmol/L) and ionized calcium (1.28 mmol/L, normal range 1.16–1.31 mmol/L), 24 h urinary excretion (3.7 mmol/L, normal range 2.5–7.5 mmol/L), 1.25 (OH) vitamin D3 (57 ng/mL, recommended levels above 50 ng/mL), and parathyroid hormone (31.1 pg/mL, normal range 12–88 pg/mL). An X-ray of the shoulder joint showed calcium deposits in the surrounding soft tissues (Figure 2A,B).

**Figure 3:**
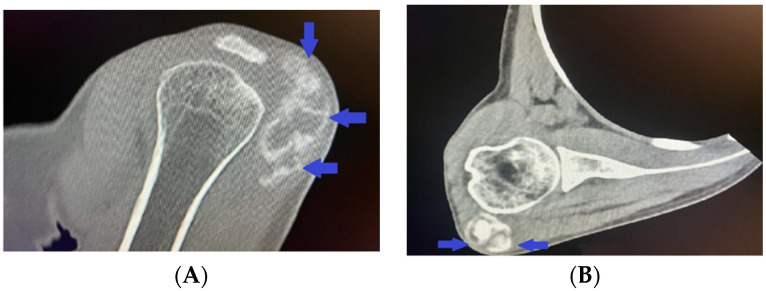
(**A**,**B**) Computed tomography with 3D reconstruction showing cloud-like lesion with soft-tissue and calcium-equivalent densitometric density in the right deltoid muscle lateral to the right humeral head and not involving adjacent bones and structures.

A hetero-dense cloud-like lesion measuring 5.22 × 1.87 cm with soft-tissue and calcium-equivalent densitometric density was seen in the right deltoid muscle at the level of the clavicle and lateral to the right humeral head, without involving neighboring bones and structures, according to spiral computed tomography with 3D reconstruction (Figure 3A,B).

**Figure 4:**
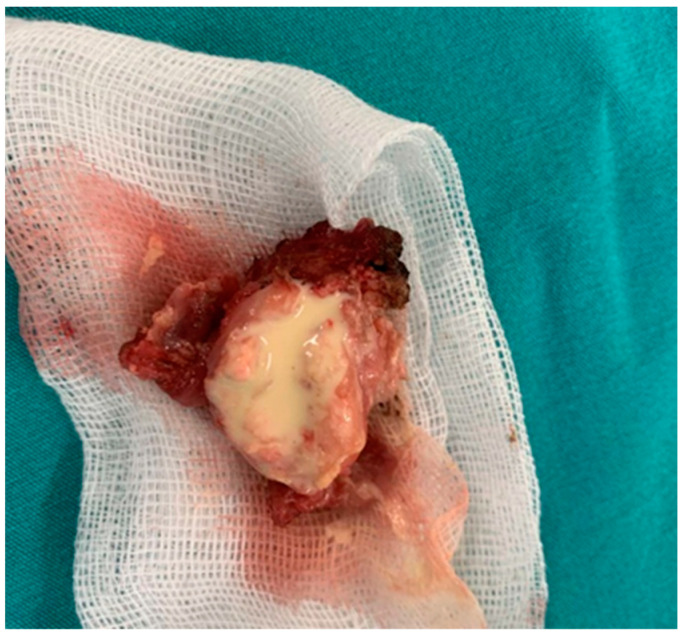
Image of the tumor that resembles a cyst with a fibrous capsule packed with thick yellowish chalky materials.

The tumor mass was removed surgically under general anesthesia. When viewed macroscopically, it resembled a cyst with a fibrous capsule containing thick chalky material that had a yellowish hue (Figure 4).

The biochemical examination found it contained calcium hydroxyapatite, calcium carbonate, and phosphate.

**Figure 5:**
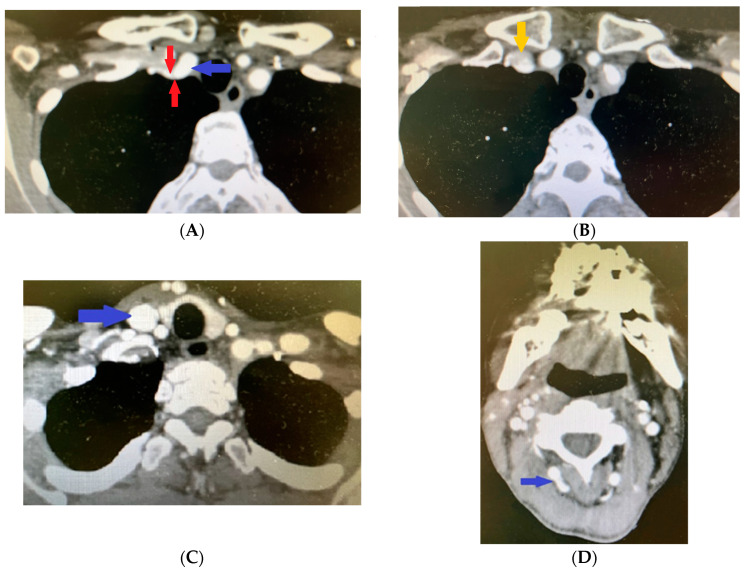
Contrast-enhanced computed tomography revealing (**A**) dilated right subclavian artery (blue arrow) followed by significant stenosis (red arrows); (**B**) parietal thrombosis in the right subclavian artery (yellow arrow); (**C**) aneurismal dilatation of the right external carotid artery (blue arrow); (**D**) fusiform dilatation of the right vertebral artery (blue arrow).

The patient saw her general practitioner in October 2021 due to swelling in the neck region and a pulsing lump on the left side of the neck. Following a clinical assessment, the physician recommended an ultrasound, which showed dilatations of 10 mm in the right external carotid artery and 12 mm in the left external carotid artery (with aneurysm) (normal range for women: 5.1 ± 1.0 mm). For these findings, contrast-enhanced brain computed tomography (CT) and angiography were performed. The results revealed the following for the extracranial vessels on the right. A dilation of 11.5 cm was seen in the right subclavian artery (normal 0.7–1.0 cm) (Figure 5A), with a subsequent segment of 30 mm with parietal thrombosis (Figure 5B) with occlusions and significant stenosis (Figure 5A).

The common carotid artery had calcium plaques in the area of bifurcation without significant stenosis. The internal carotid artery was seen with a calcium plaque in the ostium without significant stenosis but with diffuse calcium plaques in the cavernous and petrous segments and significant stenosis in the petrous segment. The external carotid artery presented postostiumally with an 8.5 to 7.5 mm aneurysmal dilatation (Figure 5C).

In addition, calcium plaques and pathological fusiform dilatations were found in the vertebral artery (Figure 5D).

**Figure 6:**
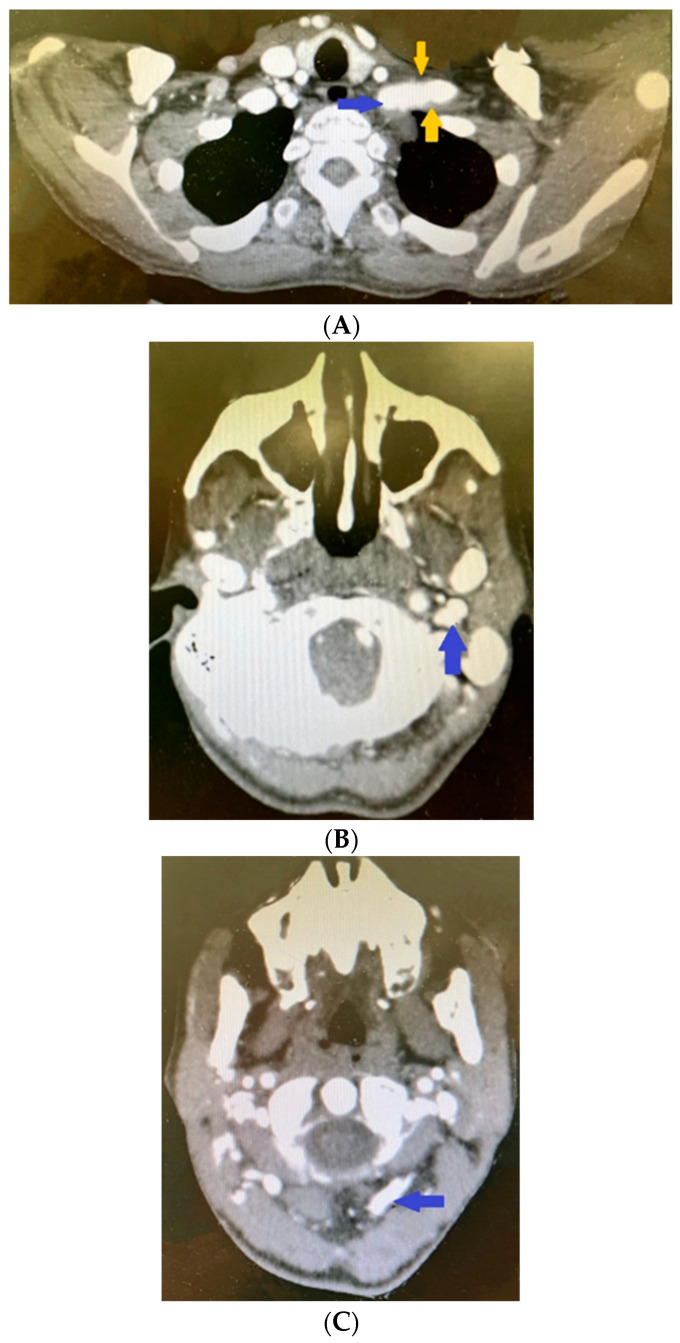
Contrast-enhanced computed tomography revealing (**A**) dilated left subclavian artery (blue arrow) and parietal thrombosis in the left subclavian artery (yellow arrows). Contrast-enhanced computed tomography revealing (**B**) aneurismal dilatation of left internal carotid artery. Contrast-enhanced computed tomography revealing (**C**) fusiform dilatation of left vertebral artery.

Significant pathological deviations were also present in the left extracranial vessels; the left subclavian artery had parietal thrombosis and a fusiform aneurysmal dilatation of 68 mm after the ostium, with maximum axial dimensions of 50 by 31 mm (Figure 6A).

The CT scan revealed calcium plaques in the bifurcation region of the common carotid artery, but no discernible stenosis. Calcium plaques also impacted the internal carotid artery, which resulted in stenosis at the pars petrosa–pars cavernosa transition and intracranial aneurysmal dilation (Figure 6B).

Immediately after the ostium, an 11.5 to 12.5 mm aneurysmal dilatation was seen in the external carotid artery. Pathological fusiform dilatation was also seen in the vertebral artery (Figure 6C).

Calcium changes were also found in the eyeballs and the retrobulbar structures, including calcifications along the inferior–posterior borders of the eyeballs. The skull bones exhibited osteosclerosis and growth. Additional alterations were seen in the prominent calcifications in the falx cerebri.

Because of the presence of aneurysmal changes and calcium plaques in the extracranial vessels, the patient’s evaluation further included coronary angiography, which revealed no stenoses or dilatation. A CT aortography with peripheral angiography of the lower extremities revealed an aneurysmally dilated lienal artery with axial dimensions of up to 8 mm (normal range 5.92 mm ± 1.2 mm) around the splenic hilus and distal dilatation of the left renal artery in the hilus to 7.7 mm (normal range in females 6.54 mm ± 0.31 mm).

Based on the imaging results, surgical intervention on the aneurysm of the left subclavian artery was performed, which included resection with a 10 mm prosthesis and reimplantation of the left vertebral artery. A stent was implanted to treat the left external carotid artery’s aneurysmal dilatation. At a later stage, the right subclavian artery was accessed retrogradely via the right common femoral artery using a catheter to pass through the occlusions and stenoses. However, this procedure was halted because the angiographic results were deemed inadequate.

Given the patient’s long history of tumor formation with soft tissue calcifications caused by disturbances in phosphorus metabolism and necessitating multiple surgical interventions, as well as the presence of vascular aneurysms, a genetic defect related to mineral metabolism was assumed. In December 2021, a laboratory in the United States completed the sequence analysis and deletion/duplication testing on 358 genes utilizing hybridization-based techniques and Illumina arrays. The investigation revealed a mutation in the FGF23 gene Exon 1, variant c.202A>G (p.Thr68Ala), and confirmed that the patient was homozygous. A heterozygous state and mutations were found in eight more genes—AMER1 variant c.1346C > G (p.Ala449Gly), CDKSRAP2 variant c.3538G > A (p.Vall180Met), CEP152 variant c.3154G > A (p.Val1052lle), MMP14 variant c.1549G > C (p.Gly517Arg), PIK3C2A variant c1910C > G (p.Pro637Arg), POP1 variant c.74G > A (p.Gly25Asp), TBX5 variant c.1343C > T (p.Pro448Leu), and WNT3A variant c.580-14C > T (Intronic). There were no mutations in GALNT3 or KL, both of which have been linked to tumoral calcinosis.

Five months after genetic testing, surgeries, and interventional procedures (in May 2022), the patient discovered that a pulsating formation in the right inguinal region had begun to grow. Following a consultation and examination by a physician, the patient had an ultrasound. The results showed that the right femoral artery aneurysm measured 2.17 by 2.12 cm, with parietal thrombosis of the lumen that was approximately ¼ of the diameter. Surgical treatment ensued, during which parietal thrombosis of the right iliac artery was discovered and thromboendarterectomy (TEA) and aneurysm resection of the right common femoral artery were performed.

**Figure 7:**
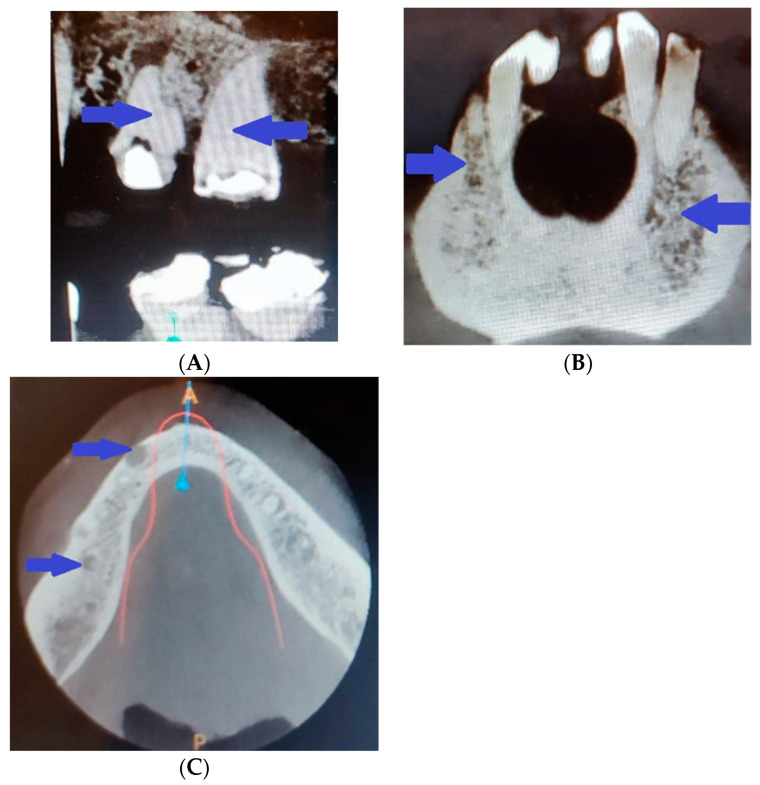
Radiography revealing (**A**) pulp calcifications and obliteration of the pulp cavity; (**B**) spongy bone structure; (**C**) some cystic masses.

In addition, there was pathological involvement of the teeth in the patient. The pulp calcifications and obliteration of the pulp cavity revealed through radiography during an in vitro procedure (Figure 7A) made treatment of the asymptomatic granulomas impossible, and five teeth were extracted. The imaging study also revealed the spongy bone structure (Figure 7B) and some cystic masses (Figure 7C).

## 3. Discussion

Hyperphosphatemic familial tumoral calcinosis (HFTC) is an extremely rare, heterogeneous genetic disorder inherited in an autosomal recessive pattern. It occurs as a result of mutations in three genes—the fibroblast growth factor-23 (FGF23), coding for a potent phosphaturic protein, KL encoding Klotho, which serves as a co-receptor for FGF23, and GALNT3, which encodes a glycosyltransferase (UDP-N-acetyl-α-D-galactosamine-polypeptide N-acetylgalactosaminyltransferase-3 (ppGalNacT3) responsible for FGF23 O-glycosylation [4]. This process occurs in the Golgi complex, where a mutation results in a lack of glycolysis (An Online Catalog of Human Genes and Genetic Disorders, OMIM:211900), allowing FGF23 to be cleaved by a proprotein convertase, most likely furin, into inactive C- and N-terminal fragments [21].

More than ten types of GALNT3 mutations have been reported, all of which result in loss of ppGalNacT3 function [22] and are associated with at least one other syndrome known as hyperostosis–hyperphosphatemia syndrome [23]. Furthermore, differences in HFTC severity and clinical manifestations are due to phenotypic heterogeneity [24].

Mutations in FGF23 (OMIM:617993) cause increased cleavage and decreased circulating intact FGF23, which is retained in the Golgi complex and secreted only as the C-terminal fragment. Affected patients have been found to have elevated levels of the C-terminal fragment [7]. Recessive variants in the KL gene (OMIM:617994) develop HFTC due to FGF23 resistance [25].

Fibroblast growth factor 23 is a peptide that is produced by osteoblasts, osteocytes [26,27], and erythroid precursor cells of the bone marrow [28]. It binds to the FGF receptor 1 (FGFR1) and its co-receptor Klotho in the proximal tubule of the kidney, where it regulates phosphorus exchange and has a phosphaturic effect. Furthermore, by stimulating 25-vitamin D-24 hydroxylase, it reduces the active form of vitamin D, 1,25-(OH)_2_-vitamin D (1,25D), whose role in phosphorus metabolism is to increase intestinal absorption [29]. FGF23 also negatively regulates parathyroid hormone secretion [30]. Mutations cause biochemical abnormalities such as hyperphosphatemia due to increased tubular reabsorption and inappropriately normal or elevated levels of 1,25D [31]. Patients with tumoral calcinosis and GLANT3 or FGF23 mutations have elevated C-terminal FGF23 fragments with low or normal FGF23 levels, whereas patients with KL mutations have elevated serum levels of both the C-terminal fragment of FGF23 and intact FGF23 [32]. Other changes that may occur as a result of the secondary high levels of 1,25D and increased calcium reabsorption include elevated blood calcium and low parathyroid hormone levels.

The disease typically manifests in the first or second decade of life [3], but in the current clinical case, it occurred at the age of 12 months. The main clinical sign is ectopic calcifications in the soft tissues around the joints [9,10], most commonly on the upper limb (shoulder and elbow), hip, foot, and wrist [11], which form tumors and sometimes necessitate surgical removal due to the involvement of joint function, as in the described case. It has been proposed that repeated microtraumas [33] or chronic pressure [34] initiate the process of forming the characteristic lesions, which begin with small hemorrhages in the periarticular tissue and initiate a foamy histiocytic response [33], which is supported by detected hemosiderin in the neighborhood [35]. As a result of the initiated healing process and the frictional forces, a neo-bursa forms, leading to the transformation of foamy histiocytes into cystic cavities via collagenolysis. Calcification gradually occurs as a result of hyperphosphatemia and hypervitaminosis D, causing the cavities to fill, become surrounded by fibrous tissue, ossify, and become relatively immobile [36].

The removed formations were biochemically analyzed, and the analysis showed that they contained primarily calcium hydroxyapatite, as well as amorphous calcium carbonate and phosphate [37]. The findings of a radiographic study included cystic periarticular lobulated formations with no bone erosion or destruction [11,38,39]. When surgical removal of the formations is imminent, computed tomography can identify “sedimentation signs”, which are defined as calcium layering with the formation of liquid–liquid levels in the cysts [39]. This is because computed tomography is primarily used to evaluate the extent of involvement of the surrounding structures [40].

Another potential phenotypic manifestation of the disease is tooth involvement, as evidenced by short, abnormal roots, pulp calcifications, and pulp cavity obliteration on a panoramic radiograph [18,41].

The progression of ectopic calcification can lead to hyperostosis, typically in the diaphyseal regions of long bones [42], involvement of skull bones, brain structures [19], eyes–eyelids and/or conjunctiva [43], the cornea [44], the retina [45], aorta, coronary arteries, and peripheral arteries [20], and the appearance of complications with the appropriate clinical picture. The literature has described a case of a family with tumoral calcinosis that required the amputation of fingers below and above the knee due to vascular calcification. The family had a proven mutation in the FGF23 gene and no other risk factors. Histological examination of the material showed calcification of the internal elastic lamina and involvement of the media. The osteocyte marker osteonectin was used, which stains capillaries, arterioles, and larger arteries [20]. Furthermore, high serum phosphorus levels are associated with an increased risk of coronary calcification in patients with normal renal function [46], and they are correlated with aortic and coronary artery calcification in patients with moderate chronic kidney disease [47], while FGF23 may shield dialysis patients from vascular calcification [48]. Additionally, messenger ribonucleic acid [mRNA] was detected in experimental settings in rats following subtotal nephrectomy and a phosphorus-rich diet, which is in line with the emergence of an osteoblast phenotype in aortic tissues [49]. The human aorta and vessels become less elastic due to calcification, which is linked to higher rates of morbidity and mortality and can be used as a separate indicator of cardiovascular morbidity and death [50].

The mechanisms by which structural changes in the vascular wall are reached—and consequently, arterial stiffness, which plays a crucial role in subsequent organ damage—are fragmentation and the subsequent degradation of elastin [51]. In turn, elastin fibers in smooth muscle cells may also be harmed by calcium deposits, which results in increased peak wall shear stress (WSS) [52]. Furthermore, a study suggests that high flow and shear stress may cause endothelial cells and smooth muscle cells to express matrix metalloproteinases 2 and 9, ultimately leading to the cell basement membrane and internal elastic lamina degradation of the arterial wall and arterial enlargement. In addition, a disproportional increase in membrane type-1- metalloproteinase and tissue inhibitor of metalloproteinase-2 was observed, which in turn might lead to matrix metalloproteinase 2 activation, causing sequential alterations [53]. Research on abdominal aortic aneurysms [54,55] provides evidence in favor of this conclusion.

Rat studies have shown that in healthy cerebral arteries, the formation of aneurysms is more likely to be caused by focal high-wall shear stress than by focal mechanical stress [56]. In this regard, it is worth noting the findings of studies that show that vascular smooth muscle cells (VSMCs) can undergo a phenotypic transition to osteoblastic, chondrocyte, and osteocyte cells, ultimately leading to calcinosis, with phosphorus hastening the process. Smooth muscle markers are lost during this transformation, and osteoblastic characteristics emerge, such as the expression of tissue-nonspecific alkaline phosphatase (Pit-1), osteocalcin, osteopontin, and osteocyte markers, including sclerostin [57,58].

In experimental studies, aortic VSMCs were extracted from mice and cultured in a growth medium containing high inorganic phosphorus (Pi). The increased Pi concentration resulted in a considerable increase in VSMC calcium deposits, as assessed using hydrochloric acid (HCL) leaching. Moreover, the VSMCs were treated with recombinant FGF23, which resulted in a significant reduction in calcium deposits and mRNA expression of osteogenic markers. In addition, VSMCs were treated with FGFR1 and FGFR3 inhibitors, which caused significant increases in calcification. Therefore, FGF-23 protects against VSMC calcification [59], and the physical association of Klotho, FGFR1, and FGFR3 is an important mechanism for preventing crucial vascular calcification [60]. These findings could serve as the foundation for a novel therapeutic strategy [59].

The woman in the presented case has a proven mutation in the FGF23 gene, which reduces FGF23 and eliminates its protective effects, and which, combined with the negative influence of hyperphosphatemia with subsequent calcinosis, results in vascular wall elastin degradation, increased arterial stiffness, and shear stress, which in turn cause expression of metalloproteinases and further destructive effects. These pathogenic mechanisms contribute significantly to structural alterations in the arterial wall, which most likely resulted in the development of multiple aneurysms. Furthermore, tumoral calcinosis is associated with cerebral and peripheral aneurysms, with only one case in a sibling found in the literature: the boy had arterial aneurysms of the brachial, iliofemoral, and coeliac axes treated surgically but died after rupture of a subclavian artery aneurysm, and the girl had carotid dysplasia and a left ophthalmic segment aneurysm that could not be treated [16].

There have been no randomized clinical trials on the treatment because the disease is extremely rare. FGF23 replacement or gene therapy is not feasible at this point in medical science. Currently, published single clinical cases or series are the primary source of data regarding the efficacy of conservative treatment [61]. Individual results vary, and assessment measures include reduced serum phosphorus levels, increased phosphate excretion, decreased tumor size, and resolution [62] achieved through limited phosphorus intake and reabsorption [63,64].

In addition to diet, phosphate-binding chelating drugs like oral aluminum hydroxide can help reduce phosphorus [64,65,66]. The combination with acetazolamide may potentially be beneficial by increasing phosphaturia [67]. In one small trial, researchers found a significant clinical and radiographic decrease in ectopic calcifications following 5 months of topical sodium thiosulfate administration [68]. When treated with ketoconazole, the authors observed a decrease in 1,25-D levels, followed by a decrease in phosphate levels [67,69]. There is no evidence that other types of agents or therapies, such as non-steroidal anti-inflammatory drugs, calcitonin, bisphosphonates, and radiation, have any relevant effect [2].

The treatment for lesions that interfere with joint function or cause pain and discomfort is surgical removal of the tumor mass [70]. It appears macroscopically as a cyst filled with a yellowish-white substance comprised of calcium hydroxyapatite crystals, calcium carbonate, and calcium phosphate [33]. Partial removal has been associated with a higher rate of recurrences than extreme removal [71,72].

A hypervascular area beyond the formation’s capsule has been found angiographically; wider excision has been suggested to cause fewer recurrences [73]. Based on scientific evidence, some authors advocate for a combination therapy strategy that includes a rigorous low-phosphate diet, medicine, and surgery [74].

The patient’s genetic testing revealed a sequence change that replaces threonine, which is neutral and polar, with alanine, which is neutral and non-polar, at codon 68 of the FGF23 protein. This variant does not appear in population datasets (Genome Aggregation Database, gnomAD, no frequency) [75]. Furthermore, this variant has not been described in the literature among persons suffering from FGF23-related disorders. Moreover, algorithms designed to predict the impact of missense changes on protein structure and function (SIFT, PolyPhen-2, and Align-GVGD) all indicate that this variant is likely to be disruptive, according to the laboratory. Given that this hitherto unknown mutation impacts the FGF 23 gene, it is highly probable that it will cause similar abnormalities linked to its function through a mechanism found in other mutations of the same gene. In this regard, the presented clinical case draws attention and provides an opportunity for new scientific research and the conducting of experiments to prove the exact mechanisms associated with the reported mutation c.202A>G (p.Thr68Ala).

The documented clinical case is the first and only one in the world to demonstrate the potential function of this sort of mutation in the FGF23 gene in the formation of a disease state, namely the hyperphosphatemic variety of tumor calcinosis. Furthermore, I predict that this homozygous mutation is connected with the disease’s aggressive course and the creation of several life-threatening arterial aneurysms. Such difficulties could theoretically be linked to any of the other eight mutations discovered in the woman, although this should be the subject of substantial genetic research. After examining the patient’s parents’ family history and origins, it was discovered that they both came from a relatively small village with around 300 persons in 1920, located about 5 km from the Greek border in the Rhodope Mountains. Most of the population of the village are Bulgarian Muslims or Bulgarian-Mohammedans. Islamization of this area occurred in the seventeenth century during the time of the Ottoman Empire. I hypothesize that variant c.202A>G (p.Thr68Ala) of the FGF23 gene mutation may have entered the village via genetic material originating from Middle Easterners, the region from which the first reports of tumoral calcinosis cases originated. Because of the isolated lifestyle of the village’s small population, I believe there is a possibility of genetic fusion between blood relations bearing the mutation (the patient’s mother and father), resulting in a homozygous state and disease manifestation in the woman.

## 4. Conclusions

The presented case is unique in that it is the first time that a pathogenic mutation variant c.202A>G (p.Thr68Ala) of the FGF23 gene has been reported, highly likely resulting in the development of the extremely rare disease hyperphosphatemic tumoral calcinosis, complicated by multiple vascular life-threatening aneurysms. Furthermore, an explanation for its connection to an area where the disease has been shown to be prevalent is put forth. It also serves as a foundation for further research on this kind of mutation, including its spread throughout Europe. In this sense, the contemporary migrant movement of individuals from the Middle East to the continent may raise the possibility of an increase in the disease’s incidence and outward presentation in tight social communities, an issue that will be the focus of future studies. The case study illustrates the necessity for further scientific investigation into genetics to find a viable cure for this uncommon, debilitating illness. The material also serves as an appeal to the global scientific community to spark interest in the one and only instance of this kind of mutation, which could alter the course of a young woman and mother’s life.

## Data Availability

The data presented in this study are available on request from the corresponding author. The data are not publicly available due to privacy/ethical reasons.

## References

[1] Inclan A., Leon P.P., Camejo M. (1943). Tumoral calcinosis. J. Am. Med. Ass..

[2] Fathi I., Sakr M. (2014). Review of tumoral calcinosis: A rare clinico-pathological entity. World J. Clin. Cases.

[3] Prince M.J., Schaefer P.C., Goldsmith R.S., Chausmer A.B. (1982). Hyperphosphatemic tumoral calcinosis: Association with elevation of serum 1,25-dihydroxycholecalciferol concentrations. Ann. Intern. Med..

[4] Sprecher E. (2010). Familial tumoral calcinosis: From characterization of a rare phenotype to the pathogenesis of ectopic calcification. J. Investig. Dermatol..

[5] Specktor P., Cooper J.G., Indelman M., Sprecher E. (2006). Hyperphosphatemic familial tumoral calcinosis caused by a mutation in GALNT3 in a European kindred. J. Hum. Genet..

[6] Topaz O., Shurman D.L., Bergman R., Indelman M., Ratajczak P., Mizrachi M., Khamaysi Z., Behar D., Petronius D., Friedman V. (2004). Mutations in GALNT3, encoding a protein involved in O-linked glycosylation, cause familial tumoral calcinosis. Nat. Genet..

[7] Benet-Pagès A., Orlik P., Strom T.M., Lorenz-Depiereux B. (2005). An FGF23 missense mutation causes familial tumoral calcinosis with hyperphosphatemia. Hum. Mol. Genet..

[8] Boyce A.M., Lee A.E., Roszko K.L., Gafni R.I. (2020). Hyperphosphatemic Tumoral Calcinosis: Pathogenesis, Clinical Presentation, and Challenges in Management. Front. Endocrinol..

[9] McClatchie S., Bremner A.D. (1969). Tumoral calcinosis--An unrecognized disease. BMJ.

[10] Lafferty F., Reynolds E., Pearson O. (1965). Tumoral calcinosis: A metabolic disease of obscure etiology. Am. J. Med..

[11] Olsen K.M., Chew F.S. (2006). Tumoral calcinosis: Pearls, polemics, and alternative possibilities. RadioGraphics.

[12] Meltzer C.C., Fishman E.K., Scott W.W. (1992). Tumoral calcinosis causing bone erosion in a renal dialysis patient. Clin. Imaging.

[13] Miyamoto K.-I., Ito M., Tatsumi S., Kuwahata M., Segawa H. (2007). New aspect of renal phosphate reabsorption: The type IIc sodium-dependent phosphate transporter. Am. J. Nephrol..

[14] Memon F., El-Abbadi M., Nakatani T., Taguchi T., Lanske B., Razzaque M.S. (2008). Does Fgf23–klotho activity influence vascular and soft tissue calcification through regulating mineral ion metabolism?. Kidney Int..

[15] Ärnlöv J., Carlsson A.C., Sundström J., Ingelsson E., Larsson A., Lind L., Larsson T.E. (2013). Serum FGF23 and risk of cardiovascular events in relation to mineral metabolism and cardiovascular pathology. Clin. J. Am. Soc. Nephrol..

[16] Adams W.M., Laitt R.D., Davies M., O’Donovan D.G. (1999). Familial tumoral calcinosis: Association with cerebral and peripheral aneurysm formation. Neuroradiology.

[17] Maiorano E., Favia G., Lacaita M.G., Limongelli L., Tempesta A., Laforgia N., Cazzolla A.P. (2014). Hyperphosphatemic familial tumoral calcinosis: Odontostomatologic management and pathological features. Am. J. Case Rep..

[18] Burkes E.J., Lyles K.W., Dolan E.A., Giammara B., Hanker J. (1991). Dental lesions in tumoral calcinosis. J. Oral. Pathol. Med..

[19] Ichikawa S., Baujat G., Seyahi A., Garoufali A.G., Imel E.A., Padgett L.R., Austin A.M., Sorenson A.H., Pejin Z., Topouchian V. (2010). Clinical variability of familial tumoral calcinosis caused by novel *GALNT3* mutations. Am. J. Med. Genet. Part. A.

[20] Shah A., Miller C.J., Nast C.C., Adams M.D., Truitt B., Tayek J.A., Tong L., Mehtani P., Monteon F., Sedor J.R. (2014). Severe vascular calcification and tumoral calcinosis in a family with hyperphosphatemia: A fibroblast growth factor 23 mutation identified by exome sequencing. Nephrol. Dial. Transplant..

[21] Tagliabracci V.S., Engel J.L., Wiley S.E., Xiao J., Gonzalez D.J., Appaiah H.N., Koller A., Nizet V., White K.E., Dixon J.E. (2014). Dynamic regulation of FGF23 by Fam20C phosphorylation, GalNAc-T3 glycosylation, and furin proteolysis. Proc. Natl. Acad. Sci. USA.

[22] Raman J., Guan Y., Perrine C.L., Gerken T.A., A. Tabak L. (2015). UDP-N-acetyl-α-d-galactosamine:polypeptide N-acetylgalactosaminyltransferases: Completion of the family tree. Glycobiology.

[23] Melhem R.E., Najjar S.S., Khachadurian A.K. (1970). Cortical hyperostosis with hyperphosphatemia: A new syndrome?. J. Pediatr..

[24] Sprecher E. (2007). Tumoral calcinosis: New insights for the rheumatologist into a familial crystal deposition disease. Curr. Rheumatol. Rep..

[25] Ichikawa S., Imel E.A., Kreiter M.L., Yu X., Mackenzie D.S., Sorenson A.H., Goetz R., Mohammadi M., White K.E., Econs M.J. (2007). A homozygous missense mutation in human KLOTHO causes severe tumoral calcinosis. J. Clin. Investig..

[26] Riminucci M., Collins M.T., Fedarko N.S., Cherman N., Corsi A., White K.E., Waguespack S., Gupta A., Hannon T., Econs M.J. (2003). FGF-23 in fibrous dysplasia of bone and its relationship to renal phosphate wasting. J. Clin. Investig..

[27] Yamamoto H., Ramos-Molina B., Lick A.N., Prideaux M., Albornoz V., Bonewald L., Lindberg I. (2016). Posttranslational processing of FGF23 in osteocytes during the osteoblast to osteocyte transition. Bone.

[28] Toro L., Barrientos V., León P., Rojas M., Gonzalez M., González-Ibáñez A., Illanes S., Sugikawa K., Abarzúa N., Bascuñán C. (2018). Erythropoietin induces bone marrow and plasma fibroblast growth factor 23 during acute kidney injury. Kidney Int..

[29] Bacchetta J., Bardet C., Prié D. (2019). Physiology of FGF23 and overview of genetic diseases associated with renal phosphate wasting. Metabolism.

[30] Ben-Dov I.Z., Galitzer H., Lavi-Moshayoff V., Goetz R., Kuro-O M., Mohammadi M., Sirkis R., Naveh-Many T., Silver J. (2007). The parathyroid is a target organ for FGF23 in rats. J. Clin. Investig..

[31] Ramnitz M.S., Gafni R.I., Collins M.T., Adam M.P., Feldman J., Mirzaa G.M., Pagon R.A., Wallace S.E., Bean L.J.H., Gripp K.W., Amemiya A. (1993–2024). Hyperphosphatemic Familial Tumoral Calcinosis. 2018 Feb 1. GeneReviews® [Internet].

[32] Razzaque M.S. (2009). The FGF23–Klotho axis: Endocrine regulation of phosphate homeostasis. Nat. Rev. Endocrinol..

[33] Smack D.P., Norton S.A., Fitzpatrick J.E. (1996). Proposal for a pathogenesis-based classification of tumoral calcinosis. Int. J. Dermatol..

[34] Thomson J.G. (1966). Calcifying collagenolysis (tumoural calcinosis). Br. J. Radiol..

[35] Pakasa N., Kalengayi R. (1997). Tumoral calcinosis: A clinicopathological study of 111 cases with emphasis on the earliest changes. Histopathology.

[36] Slavin R.E., Wen J., Kumar D., Evans E.B. (1993). Familial tumoral calcinosis. A clinical, histopathologic, and ultrastructural study with an analysis of its calcifying process and pathogenesis. Am. J. Surg. Pathol..

[37] Boskey A.L., Vigorita V.J., Sencer O., Stuchin S.A., Lane J.M. (1983). Chemical, microscopic, and ultrastructural characterization of the mineral deposits in tumoral calcinosis. Clin. Orthop. Relat. Res..

[38] Mallick S., Ahmad Z., Gupta A.K., Mathur S.R. (2013). Hyperphosphatemic tumoral calcinosis. BMJ Case Rep..

[39] Laasri K., El Houss S., Halfi I.M., Nassar I., Billah N.M. (2022). A rare case of idiopathic tumoral calcinosis: Case report. Radiol. Case Rep..

[40] Hug I., Gunçaga J. (1974). Tumoral calcinosis with sedimentation sign. Br. J. Radiol..

[41] Naikmasur V., Guttal K., Burde K., Sattur A., Nandimath K. (2008). Tumoral calcinosis with dental manifestations—A case report. Dent. Updat..

[42] Ramnitz M.S., Gourh P., Goldbach-Mansky R., Wodajo F., Ichikawa S., Econs M.J., White K.E., Molinolo A., Chen M.Y., Heller T. (2016). Phenotypic and genotypic characterization and treatment of a cohort with familial tumoral calcinosis/hyperostosis-hyperphosphatemia syndrome. J. Bone Miner. Res..

[43] Ichikawa S., Imel E.A., Sorenson A.H., Severe R., Knudson P., Harris G.J., Shaker J.L., Econs M.J. (2006). Tumoral calcinosis presenting with eyelid calcifications due to novel missense mutations in the glycosyl transferase domain of The*galnt3*gene. J. Clin. Endocrinol. Metab..

[44] Bruns D.E., Lieb W., Conway B.P., Savory J., Wills M.R., Boskey A.L. (1988). Band keratopathy and calcific lid lesions in tumoral calcinosis. Arch. Ophthalmol..

[45] McPhaul J.J., Engel F.L. (1961). Heterotopic calcification, hyperphosphatemia and angioid streaks of the retina. Am. J. Med..

[46] Foley R.N., Collins A.J., Herzog C.A., Ishani A., Kalra P.A. (2009). Serum phosphorus levels associate with coronary atherosclerosis in young adults. J. Am. Soc. Nephrol..

[47] Adeney K.L., Siscovick D.S., Ix J.H., Seliger S.L., Shlipak M.G., Jenny N.S., Kestenbaum B.R. (2009). Association of serum phosphate with vascular and valvular calcification in moderate CKD. J. Am. Soc. Nephrol..

[48] Tamei N., Ogawa T., Ishida H., Ando Y., Nitta K. (2011). Serum fibroblast growth factor-23 levels and progression of aortic arch calcification in non-diabetic patients on chronic hemodialysis. J. Atheroscler. Thromb..

[49] Román-García P., Carrillo-López N., Fernández-Martín J.L., Naves-Díaz M., Ruiz-Torres M.P., Cannata-Andía J.B. (2010). High phosphorus diet induces vascular calcification, a related decrease in bone mass and changes in the aortic gene expression. Bone.

[50] Demer L.L., Tintut Y. (2008). Vascular Calcification: Pathobiology of a multifaceted disease. Circulation.

[51] Leoncini G., Ratto E., Viazzi F., Vaccaro V., Parodi A., Falqui V., Conti N., Tomolillo C., Deferrari G., Pontremoli R. (2006). Increased ambulatory arterial stiffness index is associated with target organ damage in primary hypertension. Hypertension.

[52] Wanga S., Hibender S., Ridwan Y., van Roomen C., Vos M., van der Made I., van Vliet N., Franken R., van Riel L.A., Groenink M. (2017). Aortic microcalcification is associated with elastin fragmentation in Marfan syndrome. J. Pathol..

[53] Shoab E., Sho M., Singh T.M., Nanjob H., Komatsub M., Xua C., Masudab H., Zarins C.K. (2002). Arterial enlargement in response to high flow requires early expression of matrix metalloproteinases to degrade extracellular matrix. Exp. Mol. Pathol..

[54] Tromp G., Gatalica Z., Skunca M., Berguer R., Siegel T., Kline R.A., Kuivaniemi H. (2004). Elevated Expression of Matrix Metalloproteinase-13 in Abdominal Aortic Aneurysms. Ann. Vasc. Surg..

[55] Kataoka K., Taneda M., Asai T., Kinoshita A., Ito M., Kuroda R., Redekop G.J., Björkman J., Frösen J., Tähtinen O. (1999). Structural Fragility and Inflammatory Response of Ruptured Cerebral Aneurysms. A comparative study between ruptured and unruptured cerebral aneurysms. Stroke.

[56] Fukuda S., Hashimoto N., Naritomi H., Nagata I., Nozaki K., Kondo S., Kurino M., Kikuchi H. (2000). Prevention of rat cerebral aneurysm formation by inhibition of nitric oxide synthase. Circulation.

[57] Speer M.Y., Chien Y.-C., Quan M., Yang H.-Y., Vali H., McKee M.D., Giachelli C.M. (2005). Smooth muscle cells deficient in osteopontin have enhanced susceptibility to calcification in vitro. Cardiovasc. Res..

[58] Zhu D., Mackenzie N.C.W., Millán J.L., Farquharson C., MacRae V.E. (2011). The appearance and modulation of osteocyte marker expression during calcification of vascular smooth muscle cells. PLoS ONE.

[59] Zhu D., Mackenzie N.C., Millan J.L., Farquharson C., MacRae V.E. (2013). A protective role for FGF-23 in local defence against disrupted arterial wall integrity?. Mol. Cell. Endocrinol..

[60] Speer M.Y., Yang H.-Y., Brabb T., Leaf E., Look A., Lin W.-L., Frutkin A., Dichek D., Giachelli C.M. (2009). Smooth muscle cells give rise to osteochondrogenic precursors and chondrocytes in calcifying arteries. Circ. Res..

[61] Claramunt-Taberner D., Bertholet-Thomas A., Carlier M.-C., Dijoud F., Chotel F., Silve C., Bacchetta J. (2018). Hyperphosphatemic tumoral calcinosis caused by FGF23 compound heterozygous mutations: What are the therapeutic options for a better control of phosphatemia?. Pediatr. Nephrol..

[62] Farrow E.G., Imel E.A., White K.E. (2011). Miscellaneous non-inflammatory musculoskeletal conditions. Hyperphosphatemic familial tumoral calcinosis (FGF23, GALNT3 and αKlotho). Best. Pract. Res. Clin. Rheumatol..

[63] Gregosiewicz A., Warda E. (1989). Tumoral calcinosis: Successful medical treatment. A case report. J. Bone Jt. Surg. Am..

[64] Mozaffarian G., Lafferty F.W., Pearson O.H. (1972). Treatment of tumoral calcinosis with phosphorus deprivation. Ann. Intern. Med..

[65] Kirk T.S., A Simon M. (1981). Tumoral calcinosis. Report. of a case with successful medical management. J. Bone Jt. Surg..

[66] Davies M., Clements M.R., Mawer E.B., Freemont A.J. (1987). Tumoral calcinosis: Clinical and metabolic response to phosphorus deprivation. Q J. Med..

[67] Yamaguchi T., Sugimoto T., Imai Y., Fukase M., Fujita T., Chihara K. (1995). Successful treatment of hyperphosphatemic tumoral calcinosis with long-term acetazolamide. Bone.

[68] Jost J., Bahans C., Courbebaisse M., Tran T.-A., Linglart A., Benistan K., Lienhardt A., Mutar H., Pfender E., Ratsimbazafy V. (2016). Topical Sodium Thiosulfate: A Treatment for Calcifications in Hyperphosphatemic Familial Tumoral Calcinosis?. J. Clin. Endocrinol. Metab..

[69] Nguyen M., Boutignon H., Mallet E., Linglart A., Guillozo H., Jehan F., Garabedian M. (2010). Infantile hypercalcemia and hypercalciuria: New insights into a vitamin d-dependent mechanism and response to ketoconazole treatment. J. Pediatr..

[70] Tezelman S., Siperstein A.E., Duh Q.-Y., Clark O.H. (1993). Tumoral Calcinosis. Controversies in the etiology and alternatives in the treatment. Arch. Surg..

[71] Noyez J.F., Murphree S.M., Chen K. (1993). Tumoral calcinosis, a clinical report of eleven cases. Acta Orthop. Belg..

[72] Kisembo H., Kiguli-Malwadde E., Kawooya M.G. (2000). Tumoral calcinosis: Report of nine cases. East Afr. Med. J..

[73] Neeman Z., Wood B.J. (2003). Angiographic findings in tumoral calcinosis. Clin. Imaging.

[74] Calloway D.M., Saldaña M.J. (1993). Combined modality treatment for tumoral calcinosis. Orthop Rev..

[75] Genome Aggregation Database. https://gnomad.broadinstitute.org/about.

